# Subdiffusion of loci and cytoplasmic particles are different in compressed *Escherichia coli* cells

**DOI:** 10.1038/s42003-018-0185-5

**Published:** 2018-10-24

**Authors:** Shi Yu, Julian Sheats, Pietro Cicuta, Bianca Sclavi, Marco Cosentino Lagomarsino, Kevin D. Dorfman

**Affiliations:** 1 Department of Chemical Engineering and Materials Science, University of Minnesota—Twin Cities, 421 Washington Ave SE, Minneapolis, MN 55455 USA; 2 Cavendish Laboratory, Cambridge University, Cambridge, UK; 3 LBPA, UMR 8113 du CNRS, École Normale Supérieure Paris-Saclay, Cachan, France; 4 Génophysique/Genomic Physics Group, UMR 7238 CNRS Génomique des Microorganismes, Université Pierre et Marie Curie, 4, Place Jussieu, 75005 Paris, France; 5 Sorbonne Universités, Université Pierre et Marie Curie, 4 Place Jussieu, Paris, France; 6 IFOM Institute for Molecular Oncology, Milan, Italy; 7 Present Address: School of Chemical Engineering and Technology, China University of Mining & Technology, Xuzhou, 221116 China

## Abstract

The complex physical nature of the bacterial intracellular environment remains largely unknown, and has relevance for key biochemical and biological processes of the cell. Although recent work has addressed the role of non-equilibrium sources of activity and crowding, the consequences of mechanical perturbations are relatively less explored. Here we use a microfabricated valve system to track both fluorescently labeled chromosomal loci and cytoplasmic particles in *Escherichia coli* cells shortly after applying a compressive force, observing the response on time scales that are too sudden to allow for biochemical response from the cell. Cytoplasmic diffusion slows markedly on compression but the exponent governing the growth of the ensemble-averaged mean-squared displacement of cytoplasmic particles is unaffected. In contrast, the corresponding exponent for DNA loci changes significantly. These results suggest that DNA elasticity and nucleoid organization play a more important role in loci subdiffusion than cytoplasmic viscoelasticity under such short time scales.

## Abstract (web summary)

Shi Yu et al. show that the dynamics of fluorescently labeled DNA loci and cytoplasmic particles in *E*. *coli* respond differently to mechanical compression of the cell. These results suggest DNA elasticity is more important for determining the diffusivity of DNA loci in response to mechanical force than is cytoplasmic viscoelasticity.

## Introduction

The bacterial cytoplasm is a dense and spatially varied complex medium containing proteins, RNA, ions, and other molecules. The behavior of this medium is still poorly understood and extremely different from the dilute solutions that form the basis for our intuition about chemical reactions. As essentially all the cell content is present in the same compartment in bacteria, improving our understanding of cytoplasm behavior has very high relevance for biology. Recent reports indicate that crowding makes the cytoplasm heterogeneous and fluidized by ATP-dependent metabolic activity, exhibiting physical properties usually associated with glass-forming liquids approaching the glass transition^1^. Particle-tracking experiments using tagged RNA^2^, cytoplasmic particles^1^, and fluorescently labeled proteins bound to their chromosomal loci^3–6^ demonstrate that all of these objects undergo subdiffusion in the cytoplasm. Importantly, the exponent characterizing the subdiffusion of DNA loci is lower than what would be expected for a freely draining (Rouse) chain, which would be the expected behavior of a polymer diffusing under the strong hydrodynamic screening in a cell.

One hypothesis for the origin of the ubiquitous subdiffusion of biomolecules in *Escherichia coli* is a connection between the diffusive dynamics of the molecules and the viscoelastic properties of cytoplasm^3^. Although recent atomistic molecular dynamics simulations^7,8^ and coarse-grained simulations^9,10^ allow access to the dynamics and the heterogeneity of the cytoplasm over very short time scales, e.g., hundreds of nanoseconds, the diffusive behavior of proteins and loci on the experimental time scales of seconds cannot be computed using detailed molecular models. To circumvent this limitation, Weber et al.^11^ proposed a model combining a Rouse chain and fractional Langevin motion to capture the subdiffusion of a DNA chain in a viscoelastic medium. Subsequent work^12^ further incorporated the role of the complex folded structure^13,14^ of the chromosome embedded in a viscoelastic medium. Clearly, such a simple model cannot capture all of the details of loci diffusion in *E*. *coli*, for example, how loci mobility depends on chromosomal coordinate and subcellular localization^4^. In addition the linear subcellular arrangement of chromosomal loci with respect to their chromosomal coordinate^15^ implies an importance of intra-nucleoid interactions. Nevertheless, the appeal of the viscoelastic Rouse model^11,12^ is that it correctly predicts both the subdiffusion law and the velocity autocorrelation function of loci in the cytoplasm, suggesting that the universal physical principles of loci diffusion may arise from the intrinsic viscoelasticity of the cell. In the present contribution, we provide experimental evidence using compressed *E*. *coli* cells that indicates that this simple picture of the DNA dynamics in the bacterial cytoplasm is incomplete.

Many external stresses, such as pH change^16^, compressive force^17^, osmotic compression^18^, and glucose-starvation^19^, can stiffen cells or even promote entry into dormancy. Moreover, perturbing the cells by removing ATP removes loci subdiffusion^17^ and cytoplasmic diffusion^3,20^. However, *E*. *coli* cells can still grow and divide under compression^21^. Indeed, the rates of *E*. *coli* cell elongation, proliferation, DNA replication, and protein synthesis are not significantly changed under weak compression of ~ 5 psi (34.5 kPa), even though the *E*. *coli* cell shape changes from rod-like to pancake-like under compression^22^. Importantly, cytoplasmic diffusion is slowed down dramatically by compressive force, as evidenced by changes in FRAP (fluorescence recovery after photobleaching) measurements of *E*. *coli* cells expressing cytoplasmic fluorescent proteins with and without compression^17^. Although the mechanism causing the slowing down of cytoplasmic diffusion remains unclear, the effects of compression on *E*. *coli* cells are ideal for testing the viscoelastic Rouse model of loci diffusion^11^, as measurements made shortly after applying the compressive force directly probe the physical properties of the cell before it has time to respond biochemically.

The viscoelastic Rouse model^3,11,12^ makes a strong and testable prediction about the dynamics of cytoplasmic particles and DNA loci under compression. Explicitly, the ensemble-averaged, mean-squared displacement (MSD) of cytoplasmic particles within the cell can be quantified by

1 $${\mathrm{MSD}} = 4D_{{\mathrm{app}}}t^{\alpha}$$

where *D*_app_ is the apparent diffusion coefficient (with units of μm^2^ s^*−α*^) and the exponent *α* reveals if the diffusion is normal or anomalous. For diffusion in a viscous fluid, particles exhibit normal diffusive scaling with *α* = 1. For sufficiently long times, a segment of a polymer in the Rouse model also exhibits normal diffusion. However, for short times, the connectivity of the segments within the chain become important and the diffusivity of polymeric segments is subdiffusive with *α* = 1/2. For diffusion in a viscoelastic fluid, particles become subdiffusive *(α* < 1), where the exponent *α* is connected to the elastic memory of the fluid^11^. A key prediction of the viscoelastic Rouse model is that the ratio of the exponents is unchanged by this elastic memory; i.e., *α*_locus_ = *α*_particle_/2^11^.

In the present contribution, we do not concern ourselves with the particular quantitative connection between these exponents for the viscoelastic medium within a cell, which has been called into question^12^. Rather, we focus on the simpler question of whether compression of the cells affects the exponents *α* for cytoplasmic particles and loci in the same manner. Inasmuch as *α* is directly related to the memory kernel capturing the elastic memory of cytoplasmic diffusion^11^, our test directly probes the role of viscoelasticity on DNA loci diffusivity. On the timescale of our measurements the stress response mechanisms are not expected to be activated. Moreover, the cell wall remains intact and the cells return to their original shape once the pressure is relieved, indicating no permanent damage. Hence, this experiment corresponds to a joint measurement of the cytoplasm and chromosome viscoelasticity at short times (<2 min) after the perturbation of the cytoplasm. Our results indicate that pressure affects cytoplasmic particles and DNA loci differently, suggesting that the DNA locus mobility is more complicated than that of a Rouse chain embedded in the viscoelastic cytoplasm.

## Results

### Overall approach

**Fig. 1:**
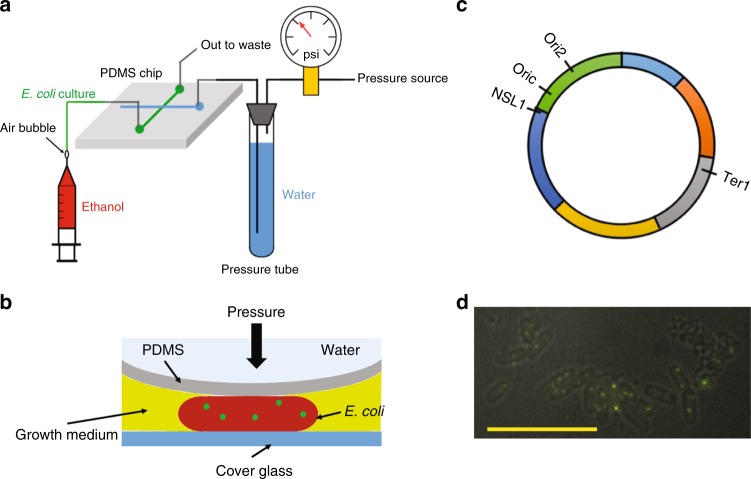
Experimental setup. **a** *E*. *coli* cells were loaded into the PDMS chip by a syringe. The control channel (blue) was pressurized and controlled by a manual pressure regulator with an airtight pressure tube to apply pressure to the PDMS valve. **b** Cartoon depicting the compression of *E*. *coli* cells using the microfabricated valve. PDMS device dimensions: ~20 mm (L) × 20 mm (W) × 10 mm (H). **c** Scheme of the loci analyzed in this work. The origin of replication is labeled by the OriC label. The chromosomal coordinates of the Ori2, Ter1, and NSL1 loci are 3,928,826, 1,056,444, and 3,739,123, respectively, at a distance of 4,943, 1,772,236, and 184,760 bp from the origin, respectively. **d** Example of tagged loci (Ori2) in *E*. *coli* cells under a compression of 15 psi. The bright dots in the image are the tagged loci. The outlines of the cell walls are distinguishable in the image. The scale bar corresponds to 10 μm

In this work, we have trapped *E*. *coli* cells under collapsed polydimethylsiloxane (PDMS) valves^23^ (Fig. 1a, b) with 200 μm wide control and flow channels^17^, allowing us to investigate the dynamics of DNA loci (Fig. 1c) and cytoplasmic particles in the flattened *E*. *coli* cells (Fig. 1d) over the relatively short time lags (~100 s) that are relevant for studying loci diffusion^4,6^. These measurements are made immediately after trapping the cells under the valves, thereby probing the physical perturbation of the cytoplasm on time scales that are too fast for the cells to respond biochemically; the typical image acquisition concludes ~2 min following the imposition of the pressure. Although both DNA loci and cytoplasmic particles exhibit non-Gaussian subdiffusion, loci motion is not slowed down significantly by cellular compression but the exponent characterizing its subdiffusion changes. In contrast, the motion of the cytoplasmic particles, which we treat as a measurement of cytoplasmic diffusion, is slowed down dramatically but the exponent is unchanged.

To probe the properties of the cytoplasm, we first measured the MSD of cytoplasmic particles. For these experiments, we used GFP-μNS particles expressed in the CJW4617 strain^1^, which itself is derived from the MG1655 *E*. *coli* strain and expresses fluorescent cytoplasmic particles that are somewhat polydisperse, with a maximum radius of 200 nm^1^. Strain CJW4617 was grown at 30 °C in M9 medium with 0.4% glycerol supplemented with casamino acids (0.5%) and 50 g/mL kanamycin (M9G). GFP-μNS particle synthesis in this strain was induced by addition of 200 μm isopropyl-d-1-thiogalactopyranoside (IPTG). After 2 h of incubation at 30 °C, induction was stopped by washing the cells into IPTG-free M9G medium^1^. We performed one set of experiments in slits with depths between 1.0 μm and 1.2 μm^24^, which are larger than the width of *E*. *coli* cells and thus provide no compression, and a second set of experiments in the device in Fig. 1b at 10 psi pressure.

### Typical mean-squared displacements

**Fig. 2:**
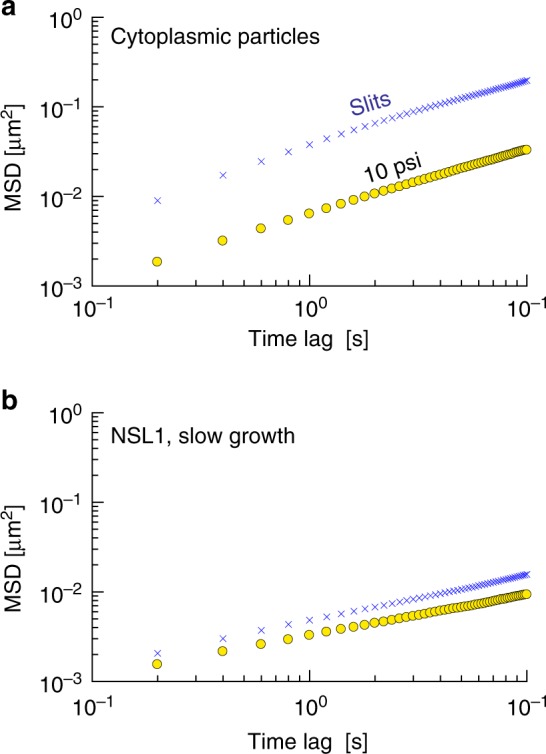
Applying pressure affects the diffusivity of cytoplasmic particles and DNA loci differently. **a** Ensemble-averaged mean-squared displacement for cytoplasmic particles^1^ confined in slits (blue multiplication symbols) and under 10 psi compression (gold circles). Linear regression to these data in Fig. S2a yields *α* = 0.75 ± 0.02 for the slits and *α* = 0.72 ± 0.005 for 10 psi compression, where the uncertainties represent 95% confidence intervals for the linear regression. **b** Equivalent data for the GFP-parB labeled NLS1 locus^25^ in slow growth media. The corresponding exponents from the linear regression in Fig. S2b are *α* = 0.51 ± 0.003 for slits and *α* = 0.45 ± 0.002 for 10 psi compression, again using 95% confidence intervals

Typical traces for cytoplasmic particles in both slits and at 10 psi applied pressure are provided in Supplementary Figure 1a–h. Figure 2a shows that the ensemble-averaged MSD obtained from thousands of such tracks (see Supplementary Table 1) is reduced by almost one order of magnitude upon compression. The dramatic reduction in MSD by compression is consistent with previous work by Okumus et al.^17^, who found that protein diffusion could be slowed by cellular compression to such an extent that one could count the individual proteins. The more important observation from Fig. 2a is that this slowdown is almost entirely owing to a change in *D*_app_ and not *α*. Linear regression to the MSD in Supplementary Figure 2a reveals that *α* = 0.75 ± 0.02 for the slits and *α* = 0.72 ± 0.005 for 10 psi compression, using 95% confidence intervals for the uncertainty. These two exponents are hardly different. In contrast, the cytoplasmic particles have apparent diffusion coefficients *D*_app_ = 0.0092 ± 0.0002 μm^2^ s^−*α*^ for slits and *D*_app_ = 0.0016 ± 0.00001 μm^2^ s^−*α*^ for 10 psi compression, again using 95% confidence intervals for the measurement uncertainty. We thus conclude from Fig. 2a that the dominant effect of compression lies in the viscous properties of the cytoplasm, not in its elastic properties.

Inasmuch as compression appears to have at most a very small effect on the elastic memory of the fluid, the viscoelastic Rouse model^11^ predicts that the exponents governing the growth of the ensemble-averaged MSD of DNA loci should also show a similarly small change under compression. However, the prototypical example for loci diffusion in a slit and under 10 psi compression provided in Fig. 2b indicates that this is not the case. These data were obtained from a modified version of the MG1655 *E*. *coli* strain^25^ where the NLS1 locus (Fig. 1c) is tagged with GFP-parB. The experiments were performed in a slow growth medium (M9 + Glu). Analysis of these MSD data in Supplementary Figure 2b reveals that the *α* exponents are now significantly different, with *α* = 0.51 ± 0.003 for slits and *α* = 0.45 ± 0.002 for 10 psi compression using 95% confidence intervals for the measurement uncertainty. We chose to only consider the MSD out to time lags of 10 seconds to avoid the influence of ballistic trajectories^6^ that tend to affect data at longer time lags and can confound the interpretation for *α*.

### Effect of pressure, DNA loci, and growth conditions

We then proceeded to determine whether this statistically significant change in *α* for DNA loci upon compression was robust to the compression pressure, DNA locus, and growth conditions. For these experiments, we continued to work with strains derived from the MG1655 *E*. *coli* strain^25^ that have been modified to express GFP-parB tagged loci (up to 120 GFP-parB molecules per locus). We considered three strains^4^, the NSL1 strain used in Fig. 2b, as well as Ori2 and Ter1 (Fig. 1c). These strains were grown in either slow growth media (M9 + Glu) and fast growth media (M9 + Glu + CAA) and the MSD for the loci were obtained under compressions of 10 psi, 15 psi, and 20 psi, as well as the uncompressed case in slits. We previously studied the MSD of a number of different loci in the absence of compression^4^. Here we have chosen the two extreme cases for the mobility to measure the full spectrum of behavior that we anticipate will be exhibited by DNA loci. We considered different growth media to control for effects of compression on cell growth rate; we know that the mobility of these DNA loci are relatively unaffected by growth rate for uncompressed cells growing on agar pads^4^.

**Fig. 3:**
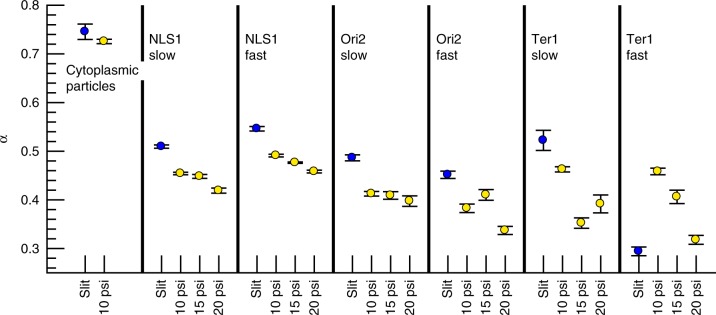
Exponents characterizing the growth of the ensemble-averaged mean-squared displacement for cytoplasmic particles and DNA loci are different under compression. The individual panels provide the exponents *α* obtained for cytoplasmic particles (Fig. 2a) as well as three different GFP-parB tagged loci (NSL1, Ori2, and Ter1; see Fig. 1c for chromosomal coordinates) under slow growth conditions (M9 + Glu) and fast growth conditions (M9 + Glu + CAA) in slits (uncompressed) and under compressions of 10 psi, 15 psi, and 20 psi. The error bars indicate 95% confidence intervals for the linear regression to the ensemble-averaged MSD data in Fig. S2; most error bars are smaller than the symbol size. Table S2 tabulates the values of *D*_app_ and *α* for each growth conditions and the uncertainty using 95% confidence intervals

We again acquired thousands of tracks at every combination of locus, growth condition and pressure (see Supplementary Table 1); several representative tracks are provided in Supplementary Figure 1i–p. For each set of conditions, we computed the ensemble-averaged MSD and fit the data to Eq. (1). Supplementary Table 2 tabulates the results for *α* and *D*_app_ for each condition, obtained from the fits in Supplementary Figure 2. Figure 3 shows that the conclusions drawn from Fig. 2 hold for all of our experimental conditions; in every case, there is a statistically significant change in *α* when the cells are compressed. In general, the trend is that increasing pressure decreases *α*. The primary anomaly lies in the data for Ter1 in fast growth media, where the exponent in slits *α* = 0.29 ± 0.009 is much lower than the other exponents and what has been observed previously for DNA loci on agar pads^4^. We do not have a complete explanation for this phenomenon. However, we do observe in Supplementary Figure 2g that Ter1 in fast growth media exhibits an anomalously large MSD at short times with an initially slow growth in the MSD, followed by more robust growth at longer time lags. In Supplementary Figure 3, we break up the MSD for Ter1 in fast growth media into two separate fits choosing the break point at time lags of 6 s. For short times, the exponent is *α* = 0.27 ± 0.01 whereas, for longer times, the exponent becomes *α* = 0.38 ± 0.02. Even at the long time lags, the growth in the MSD for Ter1 remains smaller than that under compression.

The behavior of Ter1 in fast growth media in a slit presents an intriguing puzzle that deserves further investigation. Indeed, the Ter region was previously implicated in anomalous changes in the MSD at long times from experiments on agarose pads^6^, suggesting that this macrodomain may exhibit markedly different diffusivity than other parts of the nucleoid. Nevertheless, even though the *α* exponents for compressed cells for the Ter1 locus are above that for the slit case, there is still a statistically significant change in *α* upon compression.

Overall, the *α* exponents for DNA loci from our slit device are somewhat higher than previous studies of loci subdiffusion of *E*. *coli* cells growing on agar pads^4^ and in a double-end microfluidic chemostat^5^, where the distributions in *α* were obtained from individual tracks. There are three possible sources for this discrepancy. First, the analysis of the ensemble-averaged MSD smooths out some of the heterogeneity that would be expected to result from the limited duration of the tracks. As a result, the median of a distribution of *α* obtained from individual tracks^4^ is not necessarily equal to the exponent obtained from fitting the ensemble-averaged MSD. Second, there is an adaptation time for bacteria when they are inserted into the microfluidic device^5,26^ that is longer than that for an agarose pad. Third, the *α* exponents obtained for bacteria linearized inside a microchannel tend to be somewhat higher than those on an agarose pads. In addition to the physical effects of proximity to the PDMS walls, the microfluidic systems also have glass bottoms and different access to the media, which can further affect the bacteria response.

### Non-Gaussian displacement distribution

**Fig. 4:**
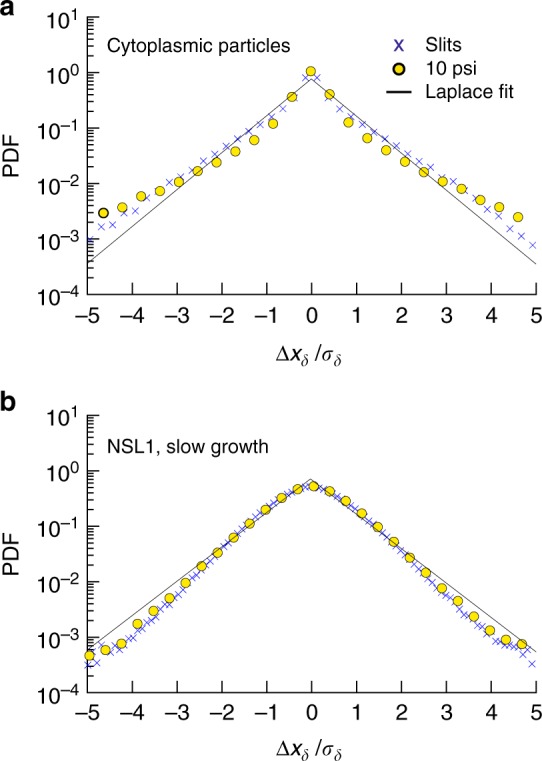
The probability density functions (PDF) of displacement distributions of cytoplasmic particles over 1 s both with and without compression have long tails. Displacement distributions over *δ* = 1 s in compressed cells at 10 psi (gold circles) and in cells packed in slits (blue multiplication symbols), scaled by the standard deviation of the displacements (*σ*_*δ*_), for **a** cytoplasmic particles and **b** NSL1 loci under slow growth conditions (M9 + Glu). The solid lines are a fit with Laplace distributions. The cells trapped by microfabricated valves or slits are randomly oriented. To expedite the analysis, we selected a fixed x-direction to be aligned with the camera sensor (i.e., not necessarily oriented along a pole of a cell) and the distribution of 1D displacement along that direction is plotted. The displacement distributions are rescaled by the standard deviation of the displacement size, *σ*_*δ*_

The different MSDs of cytoplasmic particles and loci could emerge from a fundamental change in the underlying stochastic process upon compression. To rule out this possibility, we confirmed that the step-size distributions are unaffected when the cells are compressed. Figure 4 provides a prototypical example of the displacement distribution of one locus (NSL1) in one condition (10 psi, M9 + Glu), as well as data for the cytoplasmic particles. For short times (1 s), the displacement distributions of cytoplasmic particles are controlled by the cytoplasm’s mechanical properties, whereas for long times (e.g., 10 s or more) the displacement distributions are more likely to be limited by cell membrane confinement^27^. Therefore, we only considered displacement distributions over a short time.

The non-Gaussian behavior for uncompressed cells is retained under compression, in line with the hypothesis of universality for step distributions close to Laplace distributions in this system^27^. For the DNA loci, the agreement with the Laplace distribution is good. For the cytoplasmic particles, there is a deviation in the tails that probably results from the heterogeneity in particle sizes, which is less problematic for the GFP-parB system used to label the loci.

One possible reason for the non-Gaussian diffusivity of loci is that the loci motions are driven by heterogeneous active^28^ and passive forces exerted on loci. Although the random forces felt by loci are linked to the organization of the cytoplasm, the similarity of the displacement distributions between loci and RNA–protein particles does not automatically support the idea that the underlying mechanisms of loci subdiffusion and RNA–protein particle subdiffusion are the same, as many different random processes can generate the Laplace distribution^27^. Although these are interesting insights for further investigation, the key outcome of Fig. 4 is that the step-size distribution is not affected by compression.

### Effect of pressure on cell survival

To exclude the possibility that this slowing down of cytoplasmic diffusion arises because the *E*. *coli* cells are being killed by the compressive force on cells exerted by the PDMS membrane, we performed a control experiment. After taking images, the pressure was reduced to continue to lightly trap the previously imaged cells but restore their fluidic contact with the growth medium. For the loci strains, we find that they survive up to 20 psi (see Supplementary Movies 1–4). CJW4617 cells that are first compressed at 10 psi start to regrow under the half-closed valves (see Supplementary Movies 5 and 6). Thus, for all of the data presented here, the cells survive compression.

At pressures exceeding 10 psi, we found that the CJW4617 strain expressing the cytoplasmic particles tended not to survive. All of these cells derive from the same parent strain, MG1655, and only differ in terms of the additional genomic elements inserted to express the relevant exogenous proteins^1,25^. Presumably, the toxicity of these proteins affects the ability of these otherwise similar cells to handle the stress created by the compression of the cell wall. This explanation is consistent with the tendency for the cytoplasmic particle-expressing cells to respond poorly to the stress, since their cytoplasmic particles are distributed throughout the cytoplasm and thus could interfere with a wider range of cellular activity than the parB-GFP proteins, which are localized around a single part of the nucleoid. Ideally, the different responses to pressure could be resolved by engineering a strain that expresses both the cytoplasmic particles and one of the loci tags using two different colors, and then imaging the two colors simultaneously by splitting the emitted light before reaching the camera. Although the optical problem is straightforward, the biology is non-trivial because the expression of the cytoplasmic particles is induced under different conditions than the loci labels. Moreover, even if one were to resolve the expression problem, it is possible that the presence of the cytoplasmic particles in proximity to the nucleoid could affect the loci mobility and the resolution of the loci positions (owing to spectral overlap). Such two-color experiments may provide additional insights but lie outside the scope of the present study.

## Discussion

Current models rationalize the subdiffusion of loci through a link to the cytoplasmic viscoelasticity^3,11,12^. These models make predictions that are inconsistent with our observation that the exponent characterizing the growth of the MSD changes under compression for loci but remains fixed for cytoplasmic particles. Although these theories do not make any predictions about how the cytoplasm should change upon compression, they do make predictions about what should happen to DNA loci diffusion if the cytoplasm changes. Explicitly, one central point of current theories^11,12^ is the reasonable assumption that, since the cytoplasm is the embedding medium for the chromosome, loci and cytoplasmic particles must share some diffusion properties. If this were case, then compression should affect the scaling exponent *α* for both cytoplasmic particles and DNA loci. However, we find that the changes in *α* upon compression are negligible for cytoplasmic particles and sizeable for loci, even though the underlying stochastic process, embodied by the step-size distribution in Fig. 4, is unchanged by compression.

It is unlikely that the mobility reduction of the cytoplasmic particles is specific to their large size, as the smaller particles studied by Okumus et al.^17 show the same behavior^. In addition we tend to exclude that they are blocked by a deformed nucleoid pushing them towards the cell wall, as the unvaried shape of the step-size distribution under compression in Fig. 4a and the typical size of the displacements at the smaller lags are incompatible with confinement. This leads us to conclude that the origin for the difference between the mobility of cytoplasmic particles and DNA loci lies elsewhere.

Possible reasons for the difference between the diffusivity of cytoplasmic particles and DNA loci may be hydrodynamic interactions^7,9^ or non-equilibrium effects that are ATP-dependent^1,20^. Another possibility is that the noise source for the chromosome comes selectively from this latter stable contribution from the cytoplasm, possibly through the contribution of force-generating components such as ribosomes and RNA polymerases. This could work as follows: although the ability of *E*. *coli* cells to continue to grow and divide within narrow channels^21^ or under compression^22^ implies a physiological adaptation to the geometric constraints of their environment, our measurements are too fast for the cells to react physiologically to the change in pressure and, once we release the pressure, the cells begin to grow. Thus, it appears that the only factor that has been changed dramatically by cell compression is the cytoplasmic diffusion, presumably due to loss of water that increases the cytoplasmic viscosity. Therefore, we speculate that a reason why *E*. *coli* cells change their shapes from the rod-like shape in the absence of compression to pancake-like under compression^22^ is that the imposed mechanical forces maximize the contacting interface between nucleoid and cytoplasm. As many biological reactions within cells are diffusion-limited, increasing the interfacial area between nucleoid and cytoplasm where many critical biological reactions such as transcription and translation occur^29^ is helpful to maintain the reaction rates in vivo, given that protein diffusion has been slowed down significantly^17^. It would be very informative to investigate this question using a recently reported method for visualizing the entire nucleoid^30^.

We have observed here that *E*. *coli* cells maintain their nucleoid dynamics for a compressive force up to 20 psi. This observation is in line with previous reports that *E*. *coli* cells can grow under 5 psi compression^22^ and can even get through a 300 nm wide channel^21^, which is much narrower than the *E*. *coli* width. This ability of *E*. *coli* cells to restrain the disruption of compression on their internal activities allows *E*. *coli* cells to survive external stresses. Indeed, it is plausible that much of the external compression force is exerted on the high-modulus cell membranes, so that the local structure of the nucleoid contributing to the loci subdiffusion remains unchanged. Although AFM indentation of bacteria has allowed direct measurements of the cell wall elasticity^31^, revealing how the interior environment of *E*. *coli* cells responds to compression, more experiments on cell internal structures need to be carried out to connect that body of work to our results. Although there is a body of work on the mechanics of cellular compression and its effects^22,31–33^, additional work is required to connect quantitatively our experimental results to the stress on the cells.

Our primary motivation for studying the cytoplasmic particles is to provide a set of data, obtained using exactly the same experimental protocols as those for the DNA loci, which controls for the changes to the cytoplasm upon compression. However, the data in these control experiments raise interesting questions that merit further discussion. Explicitly, our observation of the slowdown in the diffusion of the cytoplasmic particles upon compression is consistent with prior observations^17^ of the slowdown of free protein diffusion in a similar apparatus. The detailed origin of the slowdown remains elusive. Previous studies^17,34^ have speculated that the slowing down of cytoplasmic diffusion under compression emerges because the interior environment of *E*. *coli* cells becomes more crowded as water molecules are expelled by the compressive force^17^. Indeed, recent simulations of the cytoplasm^9^ reveal that a 28% bacterial cell volume decrease might result in a fivefold decrease of the diffusion coefficient of cytoplasmic proteins and an even stronger impact on the motion of larger particles.

Another possible source of slowdown is increased friction with the cell walls. We can make an estimate of the effect of shape change on loci diffusion and see whether it is sufficient to explain our results. Assuming the volume of *E*. *coli* cell remains constant under compression, we can estimate the height change of the *E*. *coli* cell owing to the compressive force, as the average area of trapped *E*. *coli* cells increases about 72% under 20 psi compression^17^. The diffusion coefficient of particles confined between two flat walls can be estimated by^35^

2 $$D_{||} = \frac{{k_{\mathrm{B}}T}}{{6\pi \eta \lambda _{||}a}} = \lambda _{||}^{ - 1}D_0 \approx D_0\left( {1 - \frac{{9a}}{{16z}}} \right)$$

where *D*_||_ is the diffusion coefficient of Brownian particle diffusing in the direction parallel to the flat walls, *k*_B_*T* is the Boltzmann factor, *η* is the fluid viscosity, and *λ*_||_ is the correction to the Stokes diffusivity for a particle of radius *a* at an average distance *z* between the flat wall and Brownian particle. Even for particles with radii up to 200 nm, which corresponds to the maximum size of the cytoplasmic particles^1^, the diffusion coefficient will only decrease by ~30% for a 42% cell height decrease, which corresponds to a 72% cell area increase in the absence of a volume change. Moreover, the slowing down of Brownian particle diffusion due to flat wall confinement is always smaller than threefold^35^. Therefore, although the drag force increase owing to the wall confinement can contribute to the slowing down of cytoplasmic particle subdiffusion, cell wall friction is not sufficient to explain the one order of magnitude decrease in Fig. 2a.

This work provides new clues for developing theoretical models of the bacterial chromosome and cytoplasm, suggesting the heterogeneity of intra-nucleoid interactions, rather than the heterogeneity of viscoelastic cytoplasm, dominates loci subdiffusion shortly after compression. The time scales of these experiments are too short for biochemical response and potentially produced unequilibrated cytoplasmic conditions that are not described by a model that treats the cytoplasm as a uniform viscoelastic medium. We suspect that the resistance of nucleoid dynamics of *E*. *coli* cells to external compressive force contributes to their survival under compression. As we already know that the bacterial chromosome is organized almost linearly according to its chromosome coordinates along the cell^15^, the stiffness of the chromosome itself rather than the mechanical properties of cytoplasm might play the most important role in loci subdiffusion. To understand better how the bacterial nucleoid responds to compression, imaging experiments on nucleoid morphology as well as loci subdiffusion in pancake-like *E*. *coli* cells under compression should be carried out. The results reported here suggest that theoretical models^3,11,14^ based on a Rouse polymer chain model are a useful starting point but do not completely capture bacterial chromosome dynamics. A deeper understanding of both the separate identity of the nucleoid from the surrounding cytoplasm and of the ATP-dependent active forces exerted on loci are required to develop a complete model to describe nucleoid dynamics.

## Methods

### Microfluidic device fabrication

Microchannels containing a microfabricated valve were produced by soft lithography in PDMS using standard microfabrication methods^23,36^. The master mold for both the flow channel and the control channel was produced by spin-coating a 12 μm AZ9260 photoresist layer onto a silicon wafer followed by ultraviolet patterning of the photoresist using contact lithography with a transparency mask defining the 200 μm wide channels. The 5 (base):1 (curing agent) PDMS (Sylgard 184, Dow Corning) replicas for the control channels were cast from this master mold. The flow channels were created by first mixing a 20 (base) :1 (curing agent) PDMS and then spin-coating the PDMS on the master at 1250 rpm for 45 seconds, which creates a circa 65 μm thick PDMS layer. Following ~55 min of baking on a hot plate at 65 °C, the PDMS membrane was aligned and bound to the 5:1 PDMS control channel layer, which had been partially cured in an oven at 75 °C for ~ 20 min. After 20 min baking on a hot plate at 75 °C to bond the control channel to the membrane layer, the completed PDMS device was peeled off from the wafer and stored in oven at 75 °C for 3 h to achieve thermal bonding. Then, this two-layer PDMS device was irreversibly plasma-bonded against a cover glass and kept in a 75 °C oven overnight. The microfabricated valves device was kept at room temperature for 1 week before use^17^. The minimum pressure required for closing the microfabricated valves is ~5 psi.

### Bacterial strains and growth conditions

For all experiments, *E. coli* cells growing in exponential phase (OD_600_ ≤ 0.3) were used. For the loci experiments, E. coli strains NSL1, Ori2, and Ter1 (Fig. 1c, ref.^4^) expressing a fluorescent parB-GFP fusion protein, originally derived from the MG1655 strain, were a gift of Frederic Boccard^25^. The bacteria were grown in LB medium with 100 μg/mL ampicillin at 37 °C overnight. The resulting *E*. *coli* cultures were diluted 200:1 in either (i) M9 media with 0.4% glucose supplemented with 50 μg/mL ampicillin (M9 + Glu) or (ii) M9 medium with 0.4% glucose supplemented with casamino acids (0.5%) and 50 μg/mL ampicillin (M9 + Glu + CAA), and were grown at 30 °C to an OD_600_ of 0.2–0.3^4,6^. We estimate that the fluorescently labeled loci typically contain ~120 parB-GFP per locus. For the cytoplasmic particles experiments, *E*. *coli* strain CJW4617^1^, also originally derived from the MG1655 strain, was grown at 30 °C in M9 medium with 0.4% glycerol supplemented with casamino acids (0.5%) and 50 μg/mL kanamycin (M9G). GFP-μNS particle synthesis in this strain was induced by addition of 200 μm IPTG. After 2 h of incubation at 30 °C, induction was stopped by washing the cells into IPTG-free M9G medium^1^. *E*. *coli* cultures were introduced into microfabricated valves (Fig. 1a) and the slits device (Fig. 1c) by hand using a syringe. Each set of experimental conditions was tested on multiple devices, typically producing 10^3^ to 10^4^ tracks (Supplementary Table 1).

### Light microscopy

Bacterial cells were imaged by an automated inverted microscope (Leica DMI-4000B) with an external Leica EL6000 light source for fluorescence imaging. Time-lapse images were taken by a Photometrics CoolSnap EZ CCD camera (Fig. 1e). The microscope and stage were controlled by Micro-manager software^37^. Fluorescent spots were detected and tracked by a custom-built script in Matlab. Details about particle-tracking can be found elsewhere^4^. The images were taken immediately after applying the pressure to the control channel. Typical lag times between applying the pressure were 10 s to ensure that the microscope was properly focused. Movies are acquired at a frame rate of five frames per second for 100 s.

### Calculation of *D*_app_ and *α*

The ensemble-averaged MSD was computed using all of the tracks reported in Supplementary Table 1, using time lags up to 10 seconds to avoid the presence of ballistic trajectories for the DNA loci^6^. The linear regression to log(MSD) versus log(Time lag) was computed in MATLAB using the fit function and the 95% confidence intervals were computed using the confint function.

### Code availability

The Matlab file used for dot tracking^4^ is available from the University of Minnesota Digital Conservancy at http://hdl.handle.net/11299/195713.

## Electronic supplementary material

SI Movie 1

SI Movie 2

SI Movie 3

SI Movie 4

SI Movie 5

SI Movie 6

Description of additional supplementary items

Supplementary file

## Data Availability

The data archive for this project is available from the Data Repository for the University of Minnesota (DRUM) at 10.13020/D6QM4N. The data appearing in the main text figures, along with Matlab scripts to generate the figures, are available from the University Digital Conservancy for the University of Minnesota at http://hdl.handle.net/11299/200485.
