# Supplementary material for: Subdiffusion of loci and cytoplasmic particles are different in compressed Escherichia coli cells
Source: Commun Biol. 2018 Oct 24;1:176. doi: 10.1038/s42003-018-0185-5 (PMC6200837; doi:10.1038/s42003-018-0185-5)
Supplement: Supplementary file 7 — Description of additional supplementary items [file 42003_2018_185_MOESM7_ESM.pdf]

**Description of additional supplementary items**

**Supplementary Movie 1:** 100 seconds movie of Ori2 strain cells under 15 psi compression in M9+Glu+CAA growth medium. The first 5 frames of the movie were overlaid by bright field images of the same spot taken before imaging the fluorescent Ori2 loci.

**Supplementary Movie 2:** About 5 hours movie of bright field images of the Ori2 strain cells at the same spot as in movie 1. Movie 2 was taken just after taking movie 1 and the pressure was reduced to ∼5 psi before movie 2 was taken.

**Supplementary Movie 3:** 100 seconds movie of Ori2 strain cells under 20 psi compression in M9+Glu+CAA growth medium. The first 5 frames of the movie were overlaid by bright field images of the same spot taken before imaging the fluorescent Ori2 loci.

**Supplementary Movie 4:** About 5 hours movie of bright field images of the Ori2 strain cells at the same spot as in movie 3. Movie 4 was taken just after taking movie 3 and the pressure was reduced to ∼5 psi before movie 4 was taken.

**Supplementary Movie 5:** 100 seconds movie of CJW4617 strain under 10 psi compression. The first 5 frames of the movie were overlaid by bright field images of the same area taken before imaging the GFP-μNS particles.

**Supplementary Movie 6:** About 5 hours movie of bright field images of CJW4617 strain cells at the same spot as in movie 5. Movie 6 was taken just after taking movie 5 and the pressure was reduced to ∼5 psi before taking movie.
