# Supplementary material for: Subdiffusion of loci and cytoplasmic particles are different in compressed Escherichia coli cells
Source: Commun Biol. 2018 Oct 24;1:176. doi: 10.1038/s42003-018-0185-5 (PMC6200837; doi:10.1038/s42003-018-0185-5)
Supplement: Supplementary file 8 — Supplementary file [file 42003_2018_185_MOESM8_ESM.pdf]

Supplementary Figures

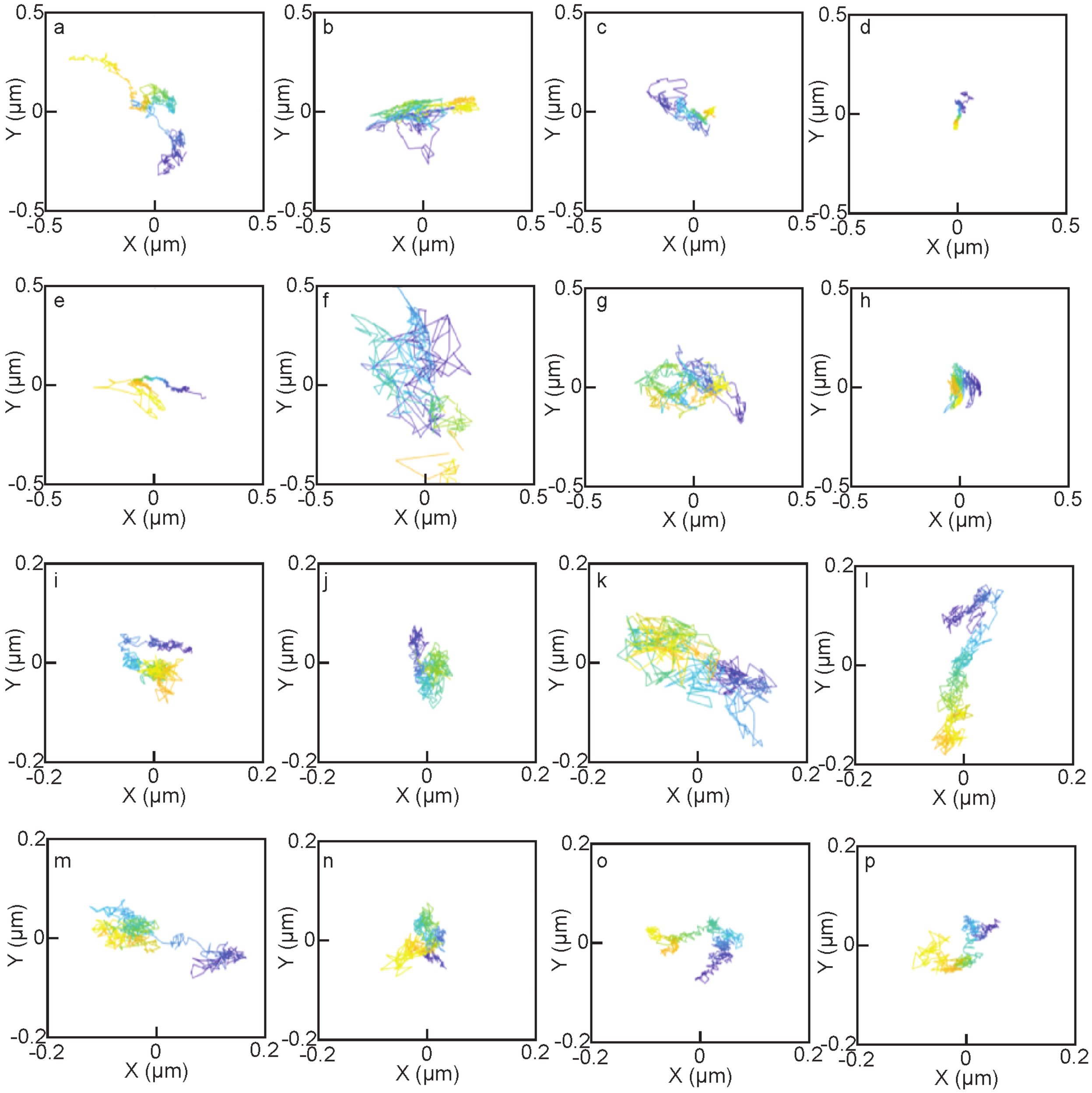

Supplementary Figure 1. Representative tracks of cytoplasmic particles and DNA loci. (a-d) Examples of cytoplasmic particles tracks color-coded by time under 10 psi compression. (e-h) Examples of cytoplasmic particles tracks color-coded by time in slits. (i-l) Examples of tagged-Ori2 loci tracks color-coded by time in slow growth conditions (M9+Glu) under 10 psi compression. (m-p) Examples of tagged-Ori2 loci tracks color-coded by time in slow growth conditions in slits.

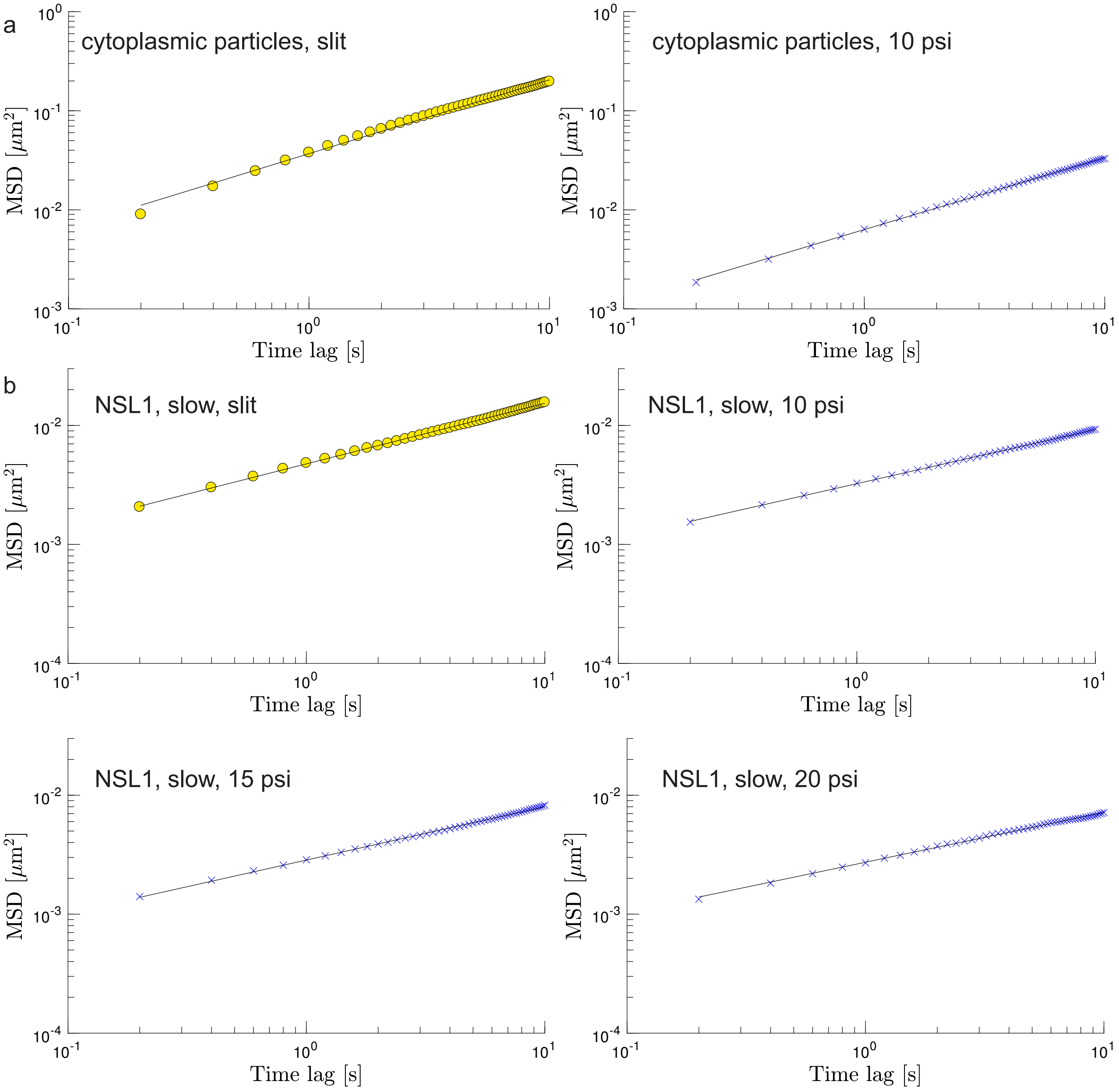
a
cytoplasmic particles, slit
cytoplasmic particles, 10 psi
b
NSL1, slow, slit
NSL1, slow, 10 psi
NSL1, slow, 15 psi
NSL1, slow, 20 psi
MSD [μm²]
Time lag [s]

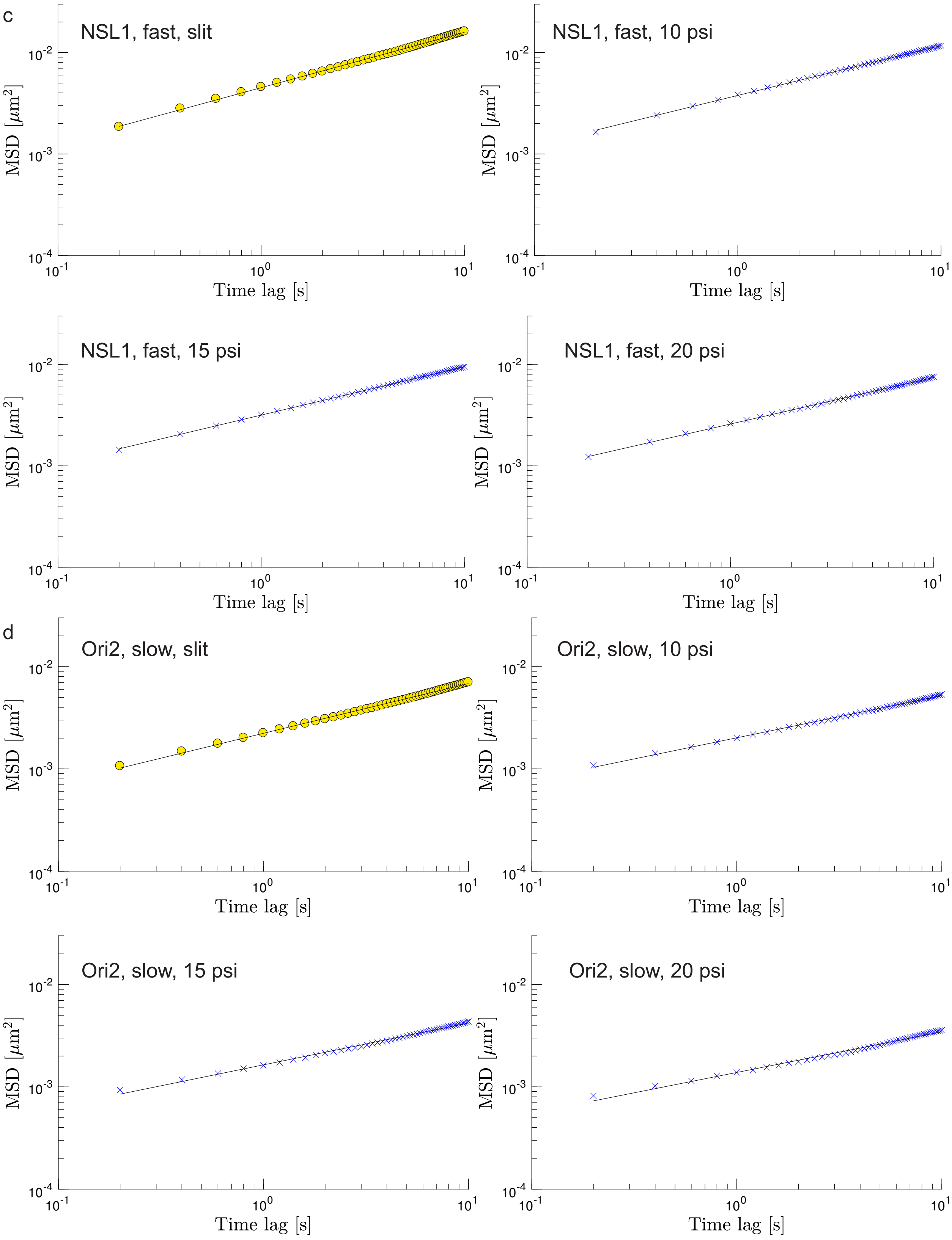
c
NSL1, fast, slit
NSL1, fast, 10 psi
NSL1, fast, 15 psi
NSL1, fast, 20 psi
d
Ori2, slow, slit
Ori2, slow, 10 psi
Ori2, slow, 15 psi
Ori2, slow, 20 psi
MSD [μm²]
Time lag [s]
10^-4
10^-3
10^-2
10^-1
10^0
10^1

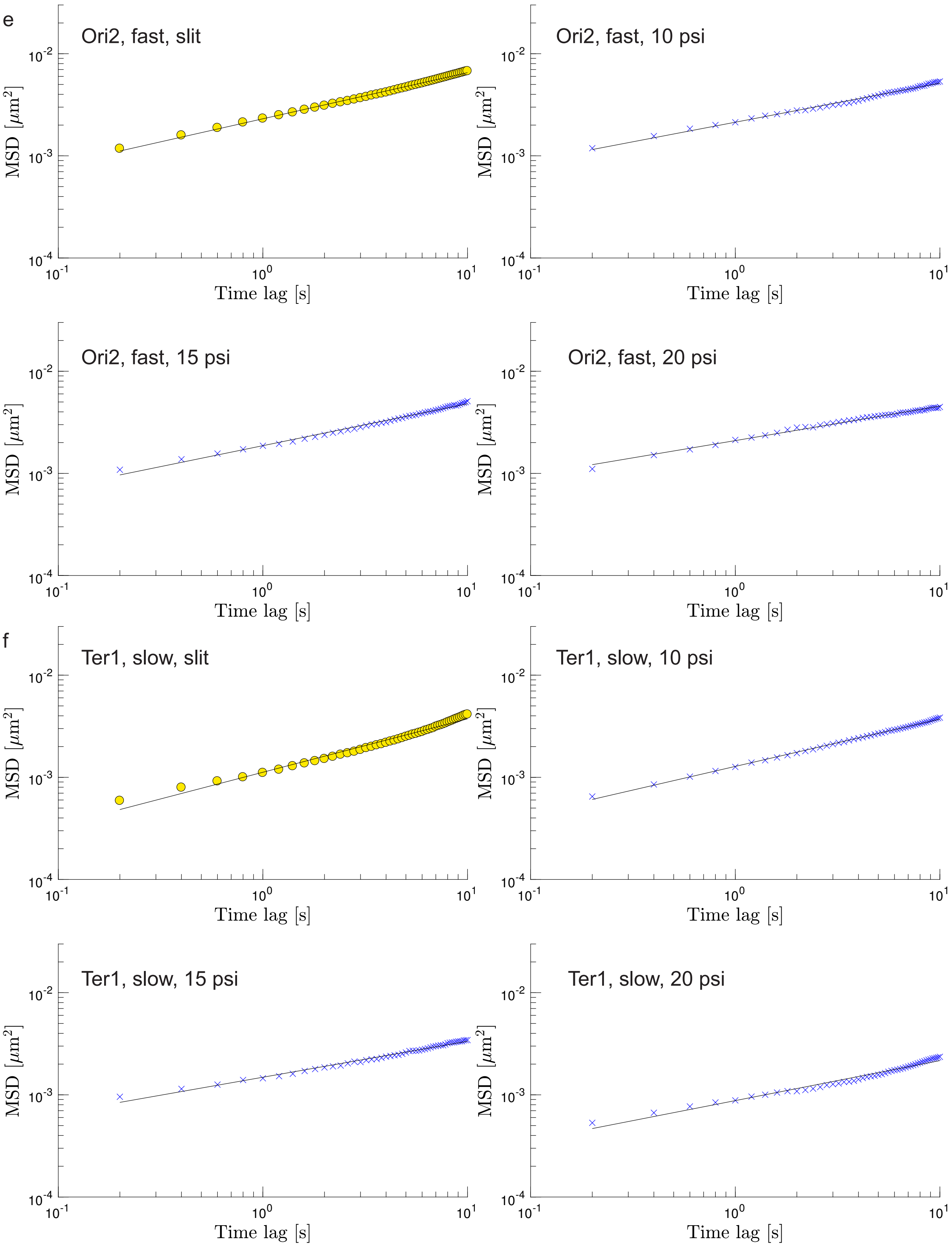

e
Ori2, fast, slit
Ori2, fast, 10 psi
Ori2, fast, 15 psi
Ori2, fast, 20 psi
f
Ter1, slow, slit
Ter1, slow, 10 psi
Ter1, slow, 15 psi
Ter1, slow, 20 psi
MSD [μm²]
Time lag [s]
10⁻²
10⁻³
10⁻⁴
10⁻¹
10⁰
10¹

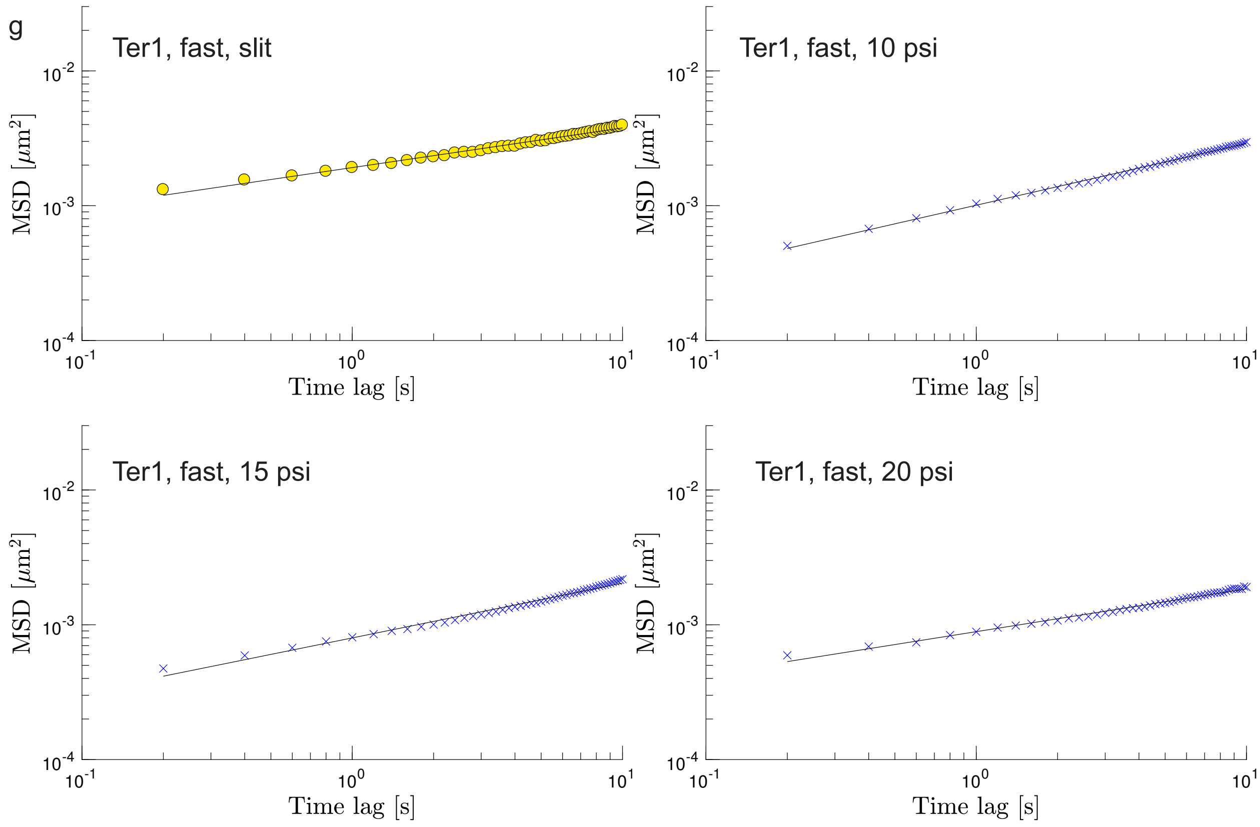

Supplementary Figure 2. Ensemble-averaged mean-squared displacement (MSD) versus time lag for (a) cytoplasmic particles (b) NSL1, M9+Glu, (c) NSL1, M9+Glu+CAA, (d) Ori2, M9+Glu, (e) Ori2, M9+Glu+CAA, (f) Ter1, M9+Glu, and (g) Ter1, M9+Glu+CAA. The solid lines are linear regressions to log(MSD) versus log(Time lag). The regression coefficients, in the form of D_{app} and α in Eq. (1), are reported in Supplementary Table 2 along with 95% confidence intervals.

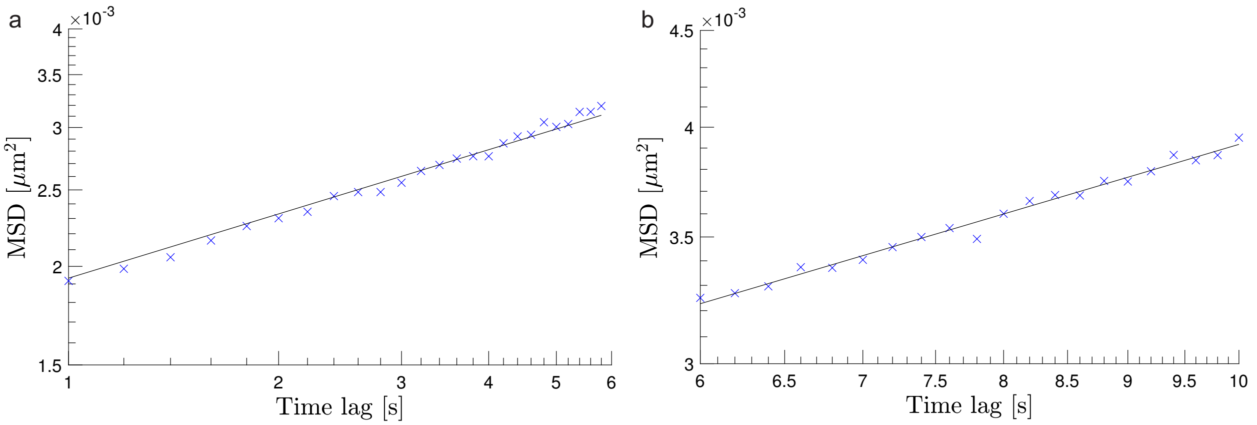

Supplementary Figure 3. Ensemble-averaged mean-squared displacement (MSD) versus time lag for Ter1 in fast growth conditions (M9+Glu+CAA) in a slit (Supplementary Figure 2g) for (a) time lags < 6 s and (b) time lags > 6s. The solid lines are linear regressions to log(MSD) versus log(Time lag). The fit for short times in (a) furnishes $\alpha = 0.27 \pm 0.01$ and $D_{\text{app}} = 0.005 \pm 0.00001$. The fit for long times in (b) furnishes $\alpha = 0.38 \pm 0.02$ and $D_{\text{app}} = 0.0004 \pm 0.00002$ $\mu\text{m}^2/\text{s}^{\alpha}$. The uncertainties are 95% confidence intervals.

Supplementary Tables

Supplementary Table 1: Number of data sets and particle tracks for each experimental condition

Strain	Chromosome coordinate	Growth medium	10 psi		15 psi		20 psi		Slits	
			Sets	Tracks	Sets	Tracks	Sets	Tracks	Sets	Tracks
Ori2	3 928 826	M9+Glu	3	1144	3	1269	3	1256	2	5979
		M9+Glu+CAA	3	1393	3	1863	3	1532	2	2780
Ter1	1 056 444	M9+Glu	2	835	2	521	2	1017	2	2514
		M9+Glu+CAA	2	765	2	1203	2	830	2	2029
NSL1	3 739 123	M9+Glu	3	1131	3	1463	3	1177	2	4119
		M9+Glu+CAA	3	5367	3	6430	3	5026	2	9092
CJW4617	—	M9G	5	1423	—	—	—	—	2	2288

Supplementary Table 2: Apparent diffusion coefficient D_{app} and exponent α for DNA loci in different growth media and different compressive pressures. The uncertainties correspond to 95% confidence intervals for the parameters obtained from linear regression to the MSD data in Supplementary Figure 2.

Strain	Growth Medium	Pressure	α	D_{app} [$\mu m^2/s^\alpha$]
NSL1	M9+Glu	Slits	0.51 ± 0.003	0.0012 ± 0.000007
NSL1	M9+Glu	10 psi	0.45 ± 0.003	0.0008 ± 0.000003
NSL1	M9+Glu	15 psi	0.45 ± 0.004	0.0007 ± 0.000005
NSL1	M9+Glu	20 psi	0.42 ± 0.005	0.0007 ± 0.000006
NSL1	M9+Glu+CAA	Slits	0.55 ± 0.004	0.0011 ± 0.000008
NSL1	M9+Glu+CAA	10 psi	0.49 ± 0.003	0.0009 ± 0.000004
NSL1	M9+Glu+CAA	15 psi	0.48 ± 0.002	0.0008 ± 0.000002
NSL1	M9+Glu+CAA	20 psi	0.46 ± 0.003	0.0006 ± 0.000003
Ori2	M9+Glu	Slits	0.49 ± 0.006	0.0006 ± 0.000006
Ori2	M9+Glu	10 psi	0.41 ± 0.005	0.0005 ± 0.000004
Ori2	M9+Glu	15 psi	0.41 ± 0.008	0.0004 ± 0.000005
Ori2	M9+Glu	20 psi	0.40 ± 0.011	0.0003 ± 0.000006
Ori2	M9+Glu+CAA	Slits	0.45 ± 0.008	0.0006 ± 0.000007
Ori2	M9+Glu+CAA	10 psi	0.38 ± 0.009	0.0005 ± 0.000008
Ori2	M9+Glu+CAA	15 psi	0.41 ± 0.011	0.0005 ± 0.000008
Ori2	M9+Glu+CAA	20 psi	0.34 ± 0.008	0.0005 ± 0.000007
Ter1	M9+Glu	Slits	0.52 ± 0.021	0.0003 ± 0.000010
Ter1	M9+Glu	10 psi	0.46 ± 0.005	0.0003 ± 0.000003
Ter1	M9+Glu	15 psi	0.35 ± 0.011	0.0004 ± 0.000007
Ter1	M9+Glu	20 psi	0.39 ± 0.018	0.0002 ± 0.000007
Ter1	M9+Glu+CAA	Slits	0.29 ± 0.009	0.0005 ± 0.000007
Ter1	M9+Glu+CAA	10 psi	0.46 ± 0.007	0.0003 ± 0.000003
Ter1	M9+Glu+CAA	15 psi	0.41 ± 0.014	0.0002 ± 0.000005
Ter1	M9+Glu+CAA	20 psi	0.32 ± 0.009	0.0002 ± 0.000003
